# Primary care physicians report high trust in and usefulness of the Stockholm drug and therapeutic committee’s list of recommended essential medicines (the ‘Wise List’)

**DOI:** 10.1007/s00228-017-2354-8

**Published:** 2017-10-23

**Authors:** Jaran Eriksen, Marie-Louise Ovesjö, Martina Vallin, Maria Juhasz-Haverinen, Eva Andersén-Karlsson, Kristina Ateva, Lars L Gustafsson, Malena Jirlow, Pia Bastholm-Rahmner

**Affiliations:** 1 Division of Clinical Pharmacology, Department of Laboratory Medicine, Karolinska Institutet, Stockholm, Sweden; 2 Department of Clinical Pharmacology, Karolinska University Hospital, 141 86 Stockholm, Sweden; 3 Department of Quality and Development, Södersjukhuset, 118 83 Stockholm, Sweden; 4 Public Healthcare Services Committee, Box 17533, 118 91 Stockholm, Sweden; 5 Department of Clinical Science and Education, Södersjukhuset, 118 83 Stockholm, Sweden; 6 Internal Medicine, Karolinska Institutet, Stockholm, Sweden; 7 Stockholm Drug and Therapeutics Committee, Public Healthcare Services Committee, Box 17533, 118 91 Stockholm, Sweden; 8 Medical Management Centre, Department of Learning, Informatics, Management and Ethics (LIME), Karolinska Institutet, 171 77 Stockholm, Sweden

**Keywords:** Adherence, Drug and therapeutic committees, Essential medicines, Guidelines, Prescribing, Rational use of medicines

## Abstract

**Purpose:**

Inappropriate use of medicines causes increased morbidity, mortality, adverse drug reactions, therapeutic failures and drug resistance as well as wastes valuable resources. Evidence-based cost-effective treatment recommendations of essential medicines are a way of avoiding these. We assessed primary care prescribers’ knowledge about and perceptions of an essential medicines formulary, as well as the reasons for adhering to the recommendations.

**Methods:**

We conducted a web based questionnaire survey targeting all physicians working in the primary healthcare of the Stockholm healthcare region (2.3 million inhabitants), regarding the knowledge of, attitudes to and usefulness of the essential medicines formulary of the Stockholm Drug and Therapeutics Committee, the so-called Wise List.

**Results:**

Of the 1862 physicians reached by our e-mail invitations, 526 (28%) participated in the survey. All but one respondent knew of the formulary, and 72% used it at least once a week when prescribing. The main reason for using the formulary was evidence-based prescribing; 97% trusted the guidelines, and almost all (98%) found the content easy to understand. At the same time, many prescribers thought that the annual changes of some recommendations were too frequent, and some felt that a national formulary would increase its trustworthiness.

**Conclusions:**

We found that the essential medicines formulary was widely used and trusted by the prescribers. The high uptake of the treatment recommendations could be due to the Stockholm Drug and Therapeutics Committee’s transparent process for developing recommendations involving respected experts and clinicians using strict criteria for handling potential conflicts of interest, feedback to prescribers, continuous medical education and minor financial incentives.

**Electronic supplementary material:**

The online version of this article (10.1007/s00228-017-2354-8) contains supplementary material, which is available to authorized users.

## Background

Inappropriate use of medicines causes increased morbidity, mortality, adverse drug reactions, therapeutic failures and drug resistance as well as being a waste of resources [1–6]. Guidelines that promote evidence-based use of cost-effective medicines could contribute to improved use of medicines. However, it is well-known that adherence to treatment recommendations is hard to achieve and has been shown to vary markedly among prescribers and between institutions [7–10].

It is especially demanding for general practitioners (GPs) to be up to date with the development within the wide range of therapeutic areas represented among their patients. It is therefore important that GPs have easy access to user-friendly, trustworthy, evidence-based and cost-effective recommendations and guidelines. However, if guidelines are not fully adopted, their potential will not be realised [11].

To improve the uptake of clinical practice guidelines, a number of different strategies have been implemented and evaluated. A recent Cochrane review concluded that implementation tools developed by the guideline committee may improve adherence, but data were too sparse to be able to assess the relative efficacy between different tools [12]. Similar findings were reported by another review that found it important with contextualisation of different strategies [7]. It is evident that the most effective implementation strategies need to be multifaceted and well-prepared and should be part of ordinary channels for strengthening the quality of care in daily practice [8]. The multifaceted approach needs to combine written and personal approaches and representation of prescribers in the development of the guidelines [8, 13].

Contrary to the well-known challenges of treatment recommendation implementation, a recent study of the adherence to the essential medicine recommendations, the so-called Wise List (See Box 1 in supplementary file), among prescribers in the Stockholm healthcare region (2.3 million inhabitants) showed that adherence to recommendations in primary care for core essential medicines was high and increased from 80 to 90% (2005 to 2015) with decreasing range in practice variation (32 to 13%) [14]. This resulted in better use of limited resources since cost-effective generic medicines were commonly first-line recommendations in the Wise List. The high adherence to the Wise List recommendations was considered to be due to the multifaceted approach that included comprehensive communication, a branding and marketing strategy with experts in a key role and integrated with a program for continuous medical education [13, 15]. The strategy also includes a system of minor financial incentives and fines related to adherence to the recommendations [16].

The aim of this study was to assess the prescribers’ knowledge about, attitudes to and perceptions of an essential medicine formulary, as well as to determine the reasons for adhering to the recommendations.

## Material and methods

### Study design and population

The method chosen for the data collection was a web survey as this was deemed to be the most suitable method to reach as many prescribers as possible. A web survey was e-mailed to the total population of 1998 physicians (including foundation-year trainees, speciality trainees, locums and general practice specialists) employed at the 215 primary healthcare centres (privately and publicly managed facilities) serving the approximately 2.3 million inhabitants in the Stockholm healthcare region in May 2015. In Stockholm, all health care is financed through public taxation with minor co-payments for prescribed medicines and outpatient visits [17]. The respondents were identified using the list of all employees registered in the Stockholm healthcare region, including name, title and place of work. The survey consisted of 20 questions about the Wise List, including knowledge of the Wise List, needs of the recommendations, frequency of use, reasons for use/non-use, user friendliness, useful sections, perceived aim of the Wise List, trust in the recommendations, perceptions of the Wise List and the Wise Advice, suggestions for improvement, preference for different editions of the Wise List, as well as demographic data for the respondent (questionnaire available as supplementary file).

### Data collection

The physicians were invited to participate in the survey by e-mail. Respondents answered the questionnaire through a link in the invitation e-mail. Participants were not offered any incentive to answer the web survey.

The questionnaire was first e-mailed to the respondents on 1 July 2015. Reminders were sent five times between August and October, after which data collection was closed in October.

**Fig. 1:**
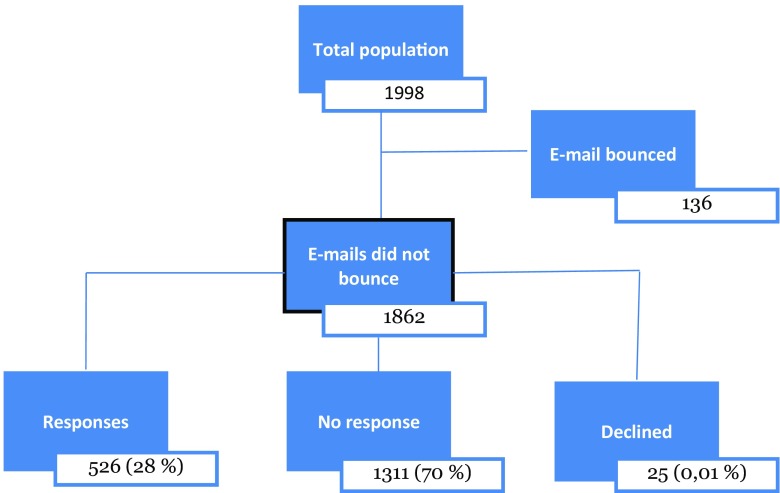
Schematic overview of the study population. Total population (*n* = 1998) based on physicians registered in the Stockholm healthcare region database as working in primary care facilities in the region

When we noted that there were no responses, despite two reminders, from the largest private primary healthcare provider in Stockholm, technical problems were suspected. Telephoning five randomly selected physicians from that service provider revealed that no one within the company had received the e-mails. It was then discovered that the e-mails were caught in the spam filters of several of the private healthcare providers. This was solved, and new e-mails were sent to the employees of these. However, it was not possible to find out how many of the e-mails had been caught in spam filters overall in the study. There were 1862 e-mail addresses from which our invitation did not bounce; from these, we received 526 responses (28%) (see Fig. 1). Information about the web survey design and data management and analysis is available in the supplementary file.

## Results

The web survey response rate was 28% (*n* = 526), i.e. more than one fourth of all physicians working in primary healthcare facilities (PHCs) in the Stockholm healthcare region. Of the respondents, 56% were female. The majority had completed their training as GPs (56%), and 30% had worked more than 20 years as a physician (Table 1).

**Table 1:** Sociodemographics of the respondents

	Respondents (*n* (%))
Total	526 (100)
Sex	
Men	204 (39)
Women	296 (56)
Did not respond	26 (5)
Years working as registered physician	
< 5	78 (15)
5–9	80 (15)
10–14	91 (17)
15–20	55 (10)
> 20	151 (30)
Not registered	49 (9)
Did not respond	22 (4)
Level of training	
Foundation year trainee	10 (2)
Specialist trainee	111 (21)
General practitioners	294 (56)
Head of clinic	62 (12)
Locum	2 (1)
Non-physician	25 (5)
Did not respond	22 (4)

Out of the 1862 individuals to whom the e-mail invitation to participate did not bounce, 526 individuals (28%) responded

When comparing the sociodemographic factors of the respondents to those of the total population of primary care physicians working in the Stockholm healthcare region, no difference between the two groups could be detected regarding the socio-economic status or geographical location of the area where they worked or the size of the healthcare facility. The respondents were also similar to the general population of primary care physicians regarding whether or not there was a person involved in the drug and therapeutics committee (DTC) work employed at the PHC.

### Reported knowledge and use of the Wise List

All but one respondent knew about the Wise List. The majority, 78%, felt that the treatment recommendations were useful, including the explanatory texts. Eighty-nine percent thought the concise, focused messages of ‘Wise piece of advice’ supported their work when prescribing medicines.

**Fig. 2:**
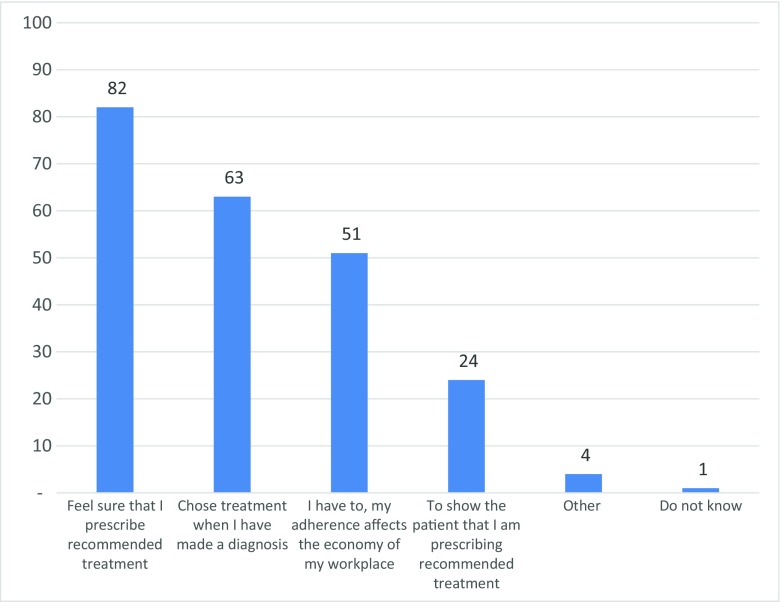
Responses to the question ‘Why do you use the wise list?’ (499 respondents). Responses in percentage. Several responses possible

Seventy-two percent of the respondents used the paper version of the Wise List at least once a week (one third of these used it daily), and 31% reported that they used the Wise List web version at least once a week. The majority, 81%, had never used the application for mobile phones or tablets. To the question ‘why do you use the Wise List?’, 82% responded that they wanted to be sure that they have prescribed the right treatment, 51% that they had to use the Wise List because it affects the economy of the facility and 24% that they use it to show the patient that they have prescribed the recommended treatment (Fig. 2).

### Reported perceived usefulness of the Wise List

The respondents stated that the three most useful therapeutic areas in the Wise List were cardiovascular diseases (64%), respiratory diseases (49%) and infections (43%). Three hundred and seven respondents explained why these were the most useful (open-ended question). The most frequent answers were the following:

- These are the most common diagnoses/treatment areas in primary health care
- Good reminder of what medicines to use and what treatment to start with
- Well-defined and relevant information
- Help to choose medicines that are not based on the pharmaceutical companies’ commercial interests

### Reasons for using the Wise List

**Fig. 3:**
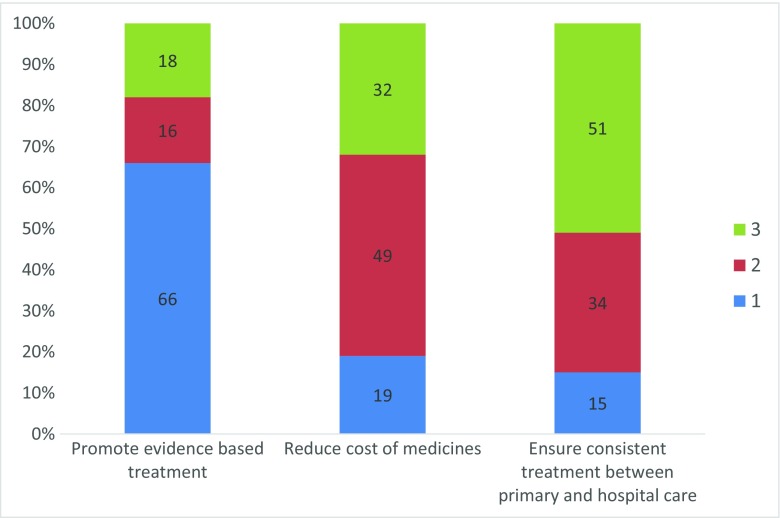
Responses to the question ‘What is your main reason for using the wise list?’ (480 respondents). Respondents could rank the three statements from 1 to 3 where 1 is the most important reason

Based on three pre-defined statements, the respondents ranked their main reasons for using the Wise List. The ranking was as follows: (1) promote evidence-based use of medicines, (2) decrease expenditure on medicines and (3) ensure consistent treatment between primary and hospital care (Fig. 3).

### Reported trust in the recommendations in the Wise List

**Fig. 4:**
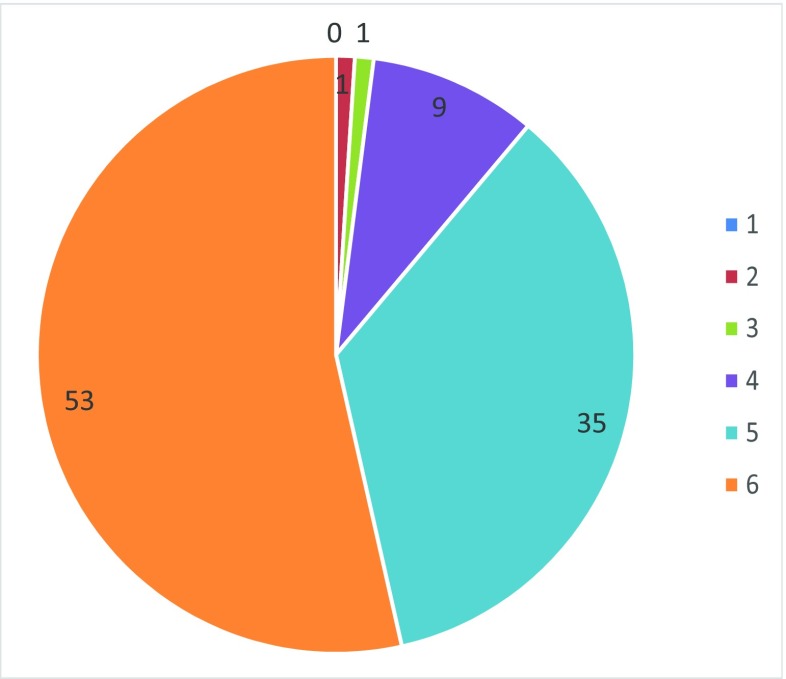
Responses to the question ‘How much do you trust the wise list?’ (491 respondents). Gradient responses where “1” = no trust and “6” = high trust

Ninety-seven percent of the respondents reported trust in the recommendations in the Wise List, of which 88% reported very high or high levels of trust (Fig. 4).

### Reported ease of use

The majority, 98%, reported that it is easy to understand the content in the Wise List and 95% that the Wise List works as support in their work. According to the amount of information in the Wise List, 78% of the respondents were satisfied with the amount of information, 14% want to have more information and 2% wanted less information in the list (6% did not know).

The paper version of the Wise List was the most used and was also considered the most user-friendly version. This was followed by the web version (www.janusinfo.se) and the application for mobile phones or tablets.

### Preferred Wise List format

In response to the question regarding what format of the Wise List they would like, almost all (89%) respondents said they wanted a printed version of the Wise List, 70% wanted a web version and 25% a mobile/tablet application (several responses possible). A larger proportion of the specialty trainees (79%) compared to the GPs (65%) wanted to have a web version of the Wise List (*p* = 0.004). This difference was even seen regarding mobile/tablet applications (43 vs. 17%, *p* < 0.0001).

### Suggestions for improvements in the Wise List

A total of 32% of the respondents suggested improvements for the Wise List. Of these, one third wrote that the Wise List did not need to be changed and that they were satisfied with both the content and the format of the current version. The suggestions for improvements are listed in Table 2 (see supplementary file).

## Discussion

We found that the primary care physicians reported high trust in the Wise List treatment recommendations and that the majority use it almost daily when prescribing medicines.

Almost all the respondents (97%) reported high trust in the Wise List recommendations. This is in concordance with earlier studies showing that trust in local DTCs seems to be an important factor for adherence to treatment recommendations [19, 20]. In our study, the respondents reported, besides high trust, a positive attitude to the recommendation and that they use the Wise List as a decision support tool in their work. Previously, we have also found that the overall adherence rate to the Wise List has increased steadily over time and that the adherence to recommendations in primary care for core medicines increased from 80 to 90% from 2005 to 2015 [14]. Already in a survey from 2005, all physicians answered that they were familiar with the Wise List concept and 81% considered the recommendations trustworthy [15]. With these results, trust seems to be an important factor for prescribers’ adherence to treatment recommendations. Prescribers in other studies have reported transparency around the structure and methods used by the DTCs as important factors for trusting recommendations [11, 19]. It has been shown that GPs want detailed background information justifying recommendations and that they were more prone to follow recommendations when they were informed about the DTCs’ process for deciding what medicines to recommend [19]. Furthermore, involvement of GPs in development of recommendations might also facilitate the trust and objectivity in DTCs’ work and might facilitate implementation of recommendations in practice [11, 13, 19]. Along these lines, some of our prescribers wanted better coordination between primary and specialist care to ensure better adherence to recommendations by all prescribers.

Transparency has been a guiding principle in the multifaceted approach of the Wise List concept to facilitate the implementation of recommendations as described [14, 15]. This approach includes some key characteristics for achieving high adherence to treatment recommendations over time, such as involving respected experts and clinicians—from both general practice and specialist care—using strict criteria for handling potential conflicts of interest, financial incentives, a wide range of activities from academic detailing to prompt electronic access to recommendations, feedback of prescribing patterns to physicians and using marketing strategies to inform the public and patients about the guidelines (Box 1—supplementary file). To achieve implementation of an effective and useful medicine formulary in the complex context of primary care is especially challenging as GPs are targeted with large amounts of information about medicines from both public and private sources [21]. The importance of using a multifaceted approach to achieve adherence to guidelines has been reported by several other researcher teams [8, 22–24].

The main aim of the Wise List is to promote evidence-based and cost-effective use of medicines [14]. There is commercial pressure on prescribers to use new, more expensive medicines even if they are not cost-effective. This is one of the reasons why the Wise List tries to counteract the prescribing of expensive medicines that do not improve the quality of treatment. Our study results show that the prescribers use the recommended medicine list primarily as a decision support tool to help them prescribe evidence-based medicines and secondly to limit costs of medicines. This is in line with earlier research showing that cost considerations of medicines are important for physicians, but safety and treatment efficacy still outrank cost concerns in the management of patients [25]. However, it is worth noting that there is a financial incentive system for prescribing medicines in the Stockholm healthcare region since 2008 [26]. For the PHCs, this incentive means that they receive a small financial bonus if their adherence to the Wise List is more than 80% and they have reflected their prescribing patterns in the yearly ‘quality report’ [16].

In our study, any opposition from prescribers towards prescribing evidence-based medicines seems related to the annual changes in the medicine formulary. Changes are made based on evidence, cost, usability and environmental aspects and may therefore change annually as the evidence base for a substance grows. When the GPs have to change a treatment to adhere to the new recommendations frequently, they feared that their patients’ trust could be reduced and thereby affect the patient-prescriber relationship negatively [23]. Another study also found lower adherence to recommendations if they were changed frequently [19]. In an earlier study, we found that the GPs are more likely to make changes in treatment to adhere to new recommendations if they perceive them to be based on factors that are important for a patient’s health rather than just related to cost [14]. Similarly, it has been reported that medical doctors and students are unaware of the costs of the medicines that they prescribe even though they state that being aware of the costs is important [27]. Many countries have introduced reforms to reduce costs of medicines [28]. In Sweden, both national and regional reforms have been introduced in order to improve adherence to medicine formularies like the Wise List [26]. These reforms include prescribing guidance and small financial incentives as described [26]. The strategy to use financial incentives has been debated, and research shows that physicians have both positive and negative attitudes to cost containment [19, 29]. The positive attitudes involve physicians’ feeling of economic responsibility for both patients and society [19] and the negative attitudes that physicians’ autonomy is threatened [22, 29]. In our study, 51% of the prescribers reported that they had to prescribe according to the Wise List because it affects the economy of their particular health centre. These findings indicate that the Wise List serves as a mix of a decision support tool for prescribing medicines as well as a financial instrument in reducing costs.

One theme mentioned by some respondents in the open-ended questions in the survey was the fact that all DTCs in Sweden have their own essential medicine formulary, and some (*n* = 12) prescribers thought that the trustworthiness would improve if the Wise List was part of a national formulary that was used across the whole country. Variations in recommended medicine lists between regions are sometimes confusing for the prescribing physicians. In a qualitative study by another Swedish group, the prescribers expressed a concern about the risk of unequal care when the treatment recommendations are produced locally, but on the other hand, they found local guidelines more trustworthy [19].

We found surprisingly few differences in attitudes to the Wise List among subgroups of respondents. The main difference was that the specialty trainees wanted a web version and mobile/tablet application of the Wise List to a larger extent (79%) than the specialist physicians (65%). It might be expected that more junior physicians with shorter work experience have a higher acceptance of using treatment guidelines and have more training from medical education in following guidelines [19, 21]. Furthermore, differences in attitudes to information about medicines have been reported where male, older, and more experienced GPs working in the private sector were more positive to industry-provided information whereas female GPs to a greater extent valued information from public authorities [21]. However, we could not see any differences between public and private facilities or male and female GPs in our study. It is possible that evidence-based medicine and a transparent academic debate have become more accepted during the years since the Stockholm Model for Wise Use of Medicines was initiated in 2000 [14, 15]. Nevertheless, direct-to-consumer pharmaceutical advertising has grown rapidly during the past decades and seems to be the most prominent way to promote prescription products directly to patients [30]. There are currently several types of advertisement to patients, but today, the most effective ones seem to be the social media campaigns which reach millions of potential consumers globally [31]. Although direct-to-consumer advertising for prescription medicines is illegal in Sweden, indirect advertisement of medicines through, e.g. magazine reports and articles, is common and sometimes encourage patients to ask their GP about a certain treatment. This trend might put a greater pressure on GPs to prescribe according to patients’ requirements rather than based on evidence or at least demand more from the prescribers with regards to explaining their decisions to patients, potentially affecting the patient-prescriber relationship [32–34].

## Methodological considerations

The response rate of 28% (*n* = 526) could raise question about the representativity of our data. Low response rates in surveys are a common challenge [35], but Visser et al. [36] argue that surveys with low response rates do not necessarily have low validity. One aspect affecting the response rate in surveys is how well-known the social context of the survey is to the respondents, i.e. who the sponsor of the survey is, and how well the respondents know the topic [37]. Concerning this aspect, we asked if someone at the respondent’s PHC had participated in DTC work or in other quality development work related to prescribing of medicines. There was no difference regarding this between the survey respondents and the general GP population in the Stockholm healthcare region. Additionally, we could not find any differences in sociodemographic factors between our respondents and the overall Stockholm primary care physician population. We therefore deem the respondents of our survey and thereby the responses representative of the primary care physician population in Stockholm.

## Conclusion

We found that the essential medicines formulary (the so-called Wise List) in our setting was widely used by prescribers. The prescribers also reported high trust in the treatment recommendations in the formulary and that evidence-based prescribing was their main reason for using it. However, the GPs were also aware that the treatment recommendations would contribute to reducing prescribing costs. The high uptake of the treatment recommendations could be due to the Stockholm DTC’s transparent process for developing recommendations involving respected experts and clinicians using strict criteria for handling potential conflicts of interest, feedback to prescribers, continuous medical education and financial incentives.

## Electronic supplementary material

ESM 1 (DOCX 101 kb)

ESM 2 (DOCX 135 kb)
