# Supplementary material for: Primary care physicians report high trust in and usefulness of the Stockholm drug and therapeutic committee’s list of recommended essential medicines (the ‘Wise List’)
Source: Eur J Clin Pharmacol. 2017 Oct 23;74(1):131–8. doi: 10.1007/s00228-017-2354-8 (PMC5748393; doi:10.1007/s00228-017-2354-8)
Supplement: Supplementary file 1 — (DOCX 101 kb) [file 228_2017_2354_MOESM1_ESM.docx]

**Supplementary file: Web survey Wise List (“Kloka Listan”)**

*The original questionnaire was in Swedish, the following is a translation made for this publication:*

Information about recommended medicines according to the Wise List are available in printed format, electronically to the web, a web application for mobile phone/tablet and as indicator marking in the patient record.

**Do you know of the Wise List?** Yes No

**What need to you have of the Wise List?**

I need both recommendations of which medicines to use, and the complementing texts in the Wise List

I only need to see what medicines are recommended in the Wise List (these can also be seen in the patient record prescribing tool)

I do not need the Wise List

Do not know/do not want to answer

Comment:

**How often do you use the Wise List?**

|  | Daily | Once a week | Once a month | Less frequently | Never |
| --- | --- | --- | --- | --- | --- |
| Printed format |  |  |  |  |  |
| Electronically through [www.janusinfo.se](http://www.janusinfo.se) |  |  |  |  |  |
| Web application for mobile/tablet |  |  |  |  |  |

**How user friendly is the printed version of the Wise List?**

1 2 3 4 5 6

Not at all Very user friendly

**How user friendly is the electronic(web) version of the Wise List at** [**www.janusinof.se**](http://www.janusinof.se) **?**

1 2 3 4 5 6

Not at all Very user friendly

**How user friendly is the web application for mobile/tablet of the Wise List?**

1 2 3 4 5 6

Not at all Very user friendly

**Why do you not use the Wise List?**

**Why do you use the Wise List?**

To be able to choose medicine for treatment after I have made the diagnosis

To make sure that I have prescribed a recommended treatment

To show the patient that I have prescribed a recommended treatment

I have to use the Wise List because it affects the economy of my health centre

Other (please specify):

Do not know/do not want to answer

**Please indicate the three most useful sections of the Wise List (***The list was in alphabetical order in the Swedish version)*:

Anaphylaxis and severe allergic reaction

Respiratory diseases

Anaemia

Anaesthesia

Children and medicines

Endocrinology

Pregnancy and breastfeeding

Gynaecology and obstetrics

Cardiovascular diseases

Dermatology and venereology

Infection

Digestive system

Neurology

Renal disease

Oncology

Osteoporosis

Preoperative care

Psychiatry

Pain and inflammation

Urology

Fluids and nutrition

Aging and medicines

Eyes

Ears

Do not know/do not want to answer

**What makes these section particularly useful?**

**What do you think about the texts in these sections (feel free to elaborate)?**

I am satisfied with the information as it is today. ___________________

I want less information. _________________________

I want more information. __________________________

Do not know/do not want to answer

**What is your main reason for using the Wise List? Rank the alternatives from 1 to 3 (1= main reason)**

Promote evidence based treatment

Decrease expenditure on medicines

Ensure consistent treatment between primary and specialised care

**How would you describe your trust in the Wise List recommendations?**

1 2 3 4 5 6

No trust High trust

**What is your perception of the Wise List and the wise advice?**

The Wise List is easy to understand

Agree completely Disagree completely Don’t know

The Wise List serves as a decision support tool in my work

Agree completely Disagree completely Don’t know

The wise advice support me in my work

Agree completely Disagree completely Don’t know

**Do you have any suggestions for the improvement of the Wise List?**

**How would you like to access the Wise List (several options possible)?**

Print format

Electronic web format

Web application for mobile/table

Other, please specify:

Do not know/do not want to answer

**Some questions about you:**

**I am**

male

female

**What best describes you?**

Foundation year trainee

Specialty trainee

Specialist physician

Head of practice

Locum physician

Other, please specify:

**Number of years as licensed physician:**

<5 years

5-9 years

10-14 years

15-20 years

>20 years

not licensed

**Are any of the employees at your health centre involved in the drug and therapeutic committee work, or any other work related to improving use of medicines?**

Yes, please specify type of work:

No

Do not know/do not want to answer
