# Supplementary material for: Primary care physicians report high trust in and usefulness of the Stockholm drug and therapeutic committee’s list of recommended essential medicines (the ‘Wise List’)
Source: Eur J Clin Pharmacol. 2017 Oct 23;74(1):131–8. doi: 10.1007/s00228-017-2354-8 (PMC5748393; doi:10.1007/s00228-017-2354-8)
Supplement: Supplementary file 2 — (DOCX 135 kb) [file 228_2017_2354_MOESM2_ESM.docx]

**Supplementary file: Detailed information not available in the manuscript**

**Box 1: The Wise List concept**

- The Wise List (“Kloka Listan” in Swedish) was developed for the whole Stockholm Healthcare Region in 2000 (the name “Wise List” was launched in 2001)
- Includes around 200 recommended core medicines for the treatment of common diseases in primary and hospital care and 100 complementary medicines for common diseases in specialised care [1]
- Covers 24 therapeutic areas
- Is a pocket-sized booklet and also available in a web-version
- Respected experts and clinicians together with clinical pharmacologists, pharmacists and nurses agree on recommendations based on review of scientific evidence using transparent criteria, including cost-effectiveness evaluation
- Includes a policy for conflict of interest with annually renewed declarations. This policy contains rules and regulations for definitions of conflict of interest and how to handle them[2]
- Includes non-pharmacological advice for several therapeutic areas. Step-wise recommendations linked to disease severity and concomitant diseases.
- The Wise List also includes around 10 “Wise piece of advice” (short, focussed messages) selected yearly to improve certain pharmacotherapeutic practices for which there are potential for improvement in quality of medicine use, e.g. “*Do not treat uncomplicated acute bronchitis with antibiotics*”
- The Wise List is communicated according to comprehensive communication, branding and marketing strategy with experts in a key role and integrated with a program for continuous medical education [3, 4]

***Web-survey design***

The survey was designed and administrated in the web-survey system Easyresearch ([www.easyresearch.se](http://www.easyresearch.se), Questback, New York, USA). It was designed with a screen-by-screen layout with one question in each screen and respondents clicking “next page” in order to proceed [5].

Some questions contained binary (yes/no) variables, some were multiple choice, some grading (1-6) type and some questions were open-ended. The appropriateness of the survey design and questions was ensured by pilot testing in 13 individuals (physicians, pharmacist, nurses and social scientists) who were familiar with the Wise List concept but were not part of the intended study population. Based on feedback received, minor adjustments were made after the pilot testing.

***Data management and analysis***

All questionnaires were saved in a database through the Easyresearch software system ([www.easyresearch.se](http://www.easyresearch.se)) and later downloaded as an excel file. Descriptive statistics were calculated for each response prior to further analysis. These descriptive findings were analysed and compared by subgroup based on demographic variables such as educational level (including foundation year trainees, speciality trainees and specialists) and work experience (number of year as a registered physician).

As not all respondents answered all questions the number of respondents varied for each question, although the non-response rate was less than 5% per question for almost all questions.

The open answers in the web-survey have been categorised using a thematic analysis and are presented with a selection of quotes from the respondents [6]. The quotes selected for this paper were translated into English.

**Table 2**: Suggestions for improvements in the Wise List from the respondents’ open answers in the survey

| **Category** | **Description of content in the category** | **Example of quotes from respondents** | **Number of respondents (n)** |
| --- | --- | --- | --- |
| Increased usability | - The usability in the paper version could be improved with for example colour tabs for the different chapters. - Simplify the website by a more intuitive interface which should make it easier to search for a medicine on line. - Integration of the electronic version of The Wise List into all medicine prescribing tools. | *“A “tab system” using different colours so that you don’t need to search for different sections but can see where they are”*  *“Better search functions. Somewhat messy when searching on janusinfo.se. Creating a separate website?”*  *“Better overview linked to the patient record, e.g. the prescribing module in TC [*patient record system used by most prescribers in Stockholm]*, maybe a visible window/button (voluntary, that would not force us to always click through more steps, a bit like Njuren* [support tool for renal function used by prescribers in Stockholm]*, indicating that you have selected a non-recommended medicine and enables you to click the window to see the recommended alternative.”* | 24 |
| Annual changes in the Wise List | - The recommended first line substances for a certain diagnosis sometimes change on an annual basis. These frequent changes could lead to decreased trust among both physicians and patients as the prescribing physicians might have difficulties to explain to the patient why the treatment changes. | *“Let the fact that a medicine has been on the Wise List be part of the consideration when updating the list. A lot of time and trust building is spent convincing patients to change medicine, especially when you have to change back to a previously used medicine.”* | 12 |
| Clear motivation when replacing a medicine | - Include a statement motivating why a medicine has been replaced on the Wise List, to make it easier to see what changes have been made* - Keep the old recommendations parallel with the new during a transitional period. | *“I would like to see motivations for reasons why a substance has been changed from one year to another, e.g. clickable links next to the new substance.”* | 12 |
| Dosage and treatment length | - More information and advice on dose for a recommended medicine and recommended treatment length. | *“suggested dosing and advice on treatment initiation would help.”*  *“more information regarding dosing and how long to continue treatment.”* | 12 |
| One national list on recommended medicines | - The Wise List trustworthiness would improve if it was part of a national list with recommended medicines for the whole country. Variations in recommended medicines lists between regions are sometimes confusing for the prescribing physicians. | *“The trustworthiness would improve if the list was nationwide. When the lists vary within the country it seems arbitrary what substance has been recommended in a healthcare region.”* | 12 |
| Coordination between primary and secondary care among recommended medicines | - When a medicine is prescribed by a physician in specialist care it is often difficult for the GP to change it to a substance recommended on the Wise List. The respondents therefore wanted a better developed coordination between primary and secondary care. | *“More information (and maybe more external pressure) is needed to ensure specialist care follows the recommendations. The primary care is often held responsible for treatments that are not according to the Wise List, although in many cases a physician in specialist care has prescribed the “wrong” substance and the primary care takes over responsibility for the patient.* | 5 |
| The system with “incentives and fines” hampers therapeutic principles | - It should be specified when a medicine is recommended due to economic reasons instead of therapeutic principles. Some physicians felt that the system with economic incentives and fines hampers the idea that that the Wise List is based on therapeutic principals. | *“Fewer individual substance recommendations, more therapeutic principles, in cases when price is the only reason a substance has been selected this should be stated.”* | 4 |
| Herbal medicines | - Include information on herbal medicines’ interactions with recommended medicines. |  | 3 |
| Devices for drug delivery and disease monitoring | - Include list of the drug delivery and diseas monitoring devices, such as nebulisers, injection tools, test sticks et., available for the patients. |  | 3 |

*****this already exists in the web version of the Wise List, but does not seem to be known by all respondents.

**References**:

1. Eriksen, J., et al., *High adherence to the 'Wise List' treatment recommendations in Stockholm: a 15-year retrospective review of a multifaceted approach promoting rational use of medicines.* BMJ Open, 2017. **7**(4): p. e014345.

2. Stockholm County Counsil, D.T.C. *Jävspolicy för läkemedelskommittéorganisationen i Stockholms läns landsting (SLL)*. 2011 [cited 2016; Available from: <http://www.janusinfo.se/Global/SLK/javspolicy_slk_2011_2.pdf>.

3. Gustafsson, L.L., et al., *The 'wise list'- a comprehensive concept to select, communicate and achieve adherence to recommendations of essential drugs in ambulatory care in Stockholm.* Basic Clin Pharmacol Toxicol, 2011. **108**(4): p. 224-33.

4. Bjorkhem-Bergman, L., et al., *Interface management of pharmacotherapy. Joint hospital and primary care drug recommendations.* Eur J Clin Pharmacol, 2013. **69 Suppl 1**: p. 73-8.

5. Peytchev, A., et al., *Web survey design: Paging versus scrolling.* Public Opinion Quarterly, 2006. **70**(4): p. 596-607.

6. Patton, M.Q., *Qualitative Research and Evaluation Methods*. 3rd edition ed. 2002, Thousand Oaks, California: Sage Publications.
